# Totally laparoscopic resection using delta-shaped anastomosis of jejunal leiomyosarcoma with intussusception at the angle of Treitz: a case report

**DOI:** 10.1186/s40792-022-01541-3

**Published:** 2022-09-26

**Authors:** Kenichi Nakamura, Susumu Shibasaki, Seiji Yamada, Kazumitsu Suzuki, Akiko Serizawa, Shingo Akimoto, Masaya Nakauchi, Tsuyoshi Tanaka, Kazuki Inaba, Ichiro Uyama, Koichi Suda

**Affiliations:** 1 Department of Surgery, Fujita Health University, 1-98 Dengakugakubo, Kutsukake, Toyoake, Aichi 470-1192 Japan; 2 Department of Diagnostic Pathology, Fujita Health University, 1-98 Dengakugakubo, Kutsukake, Toyoake, Aichi 470-1192 Japan; 3 Advanced Robotic and Endoscopic Surgery, Fujita Health University, 1-98 Dengakugakubo, Kutsukake, Toyoake, Aichi 470-1192 Japan; 4 Collaborative Laboratory for Research and Development in Advanced Surgical Technology, Fujita Health University, 1-98 Dengakugakubo, Kutsukake, Toyoake, Aichi 470-1192 Japan

**Keywords:** Small bowel tumor, Leiomyosarcoma, Laparoscopic surgery, Mesenchymal tumors

## Abstract

**Background:**

A leiomyosarcoma of the gastrointestinal tract is extremely rare. We report a case of jejunal leiomyosarcoma with intestinal intussusception at the angle of Treitz that was successfully treated with laparoscopic resection followed by intracorporeal reconstruction using a delta-shaped anastomosis.

**Case presentation:**

A 54-year-old man was referred to our hospital due to fatigue and loss of appetite. Blood tests showed anemia. Enteroscopy and subsequent enterography using meglumine sodium amidotrizoate showed easily hemorrhagic tumor (10 cm in diameter) in the jejunum just beyond the angle of Treitz. Contrast-enhanced computed tomography revealed jejunojejunal intussusception. Histopathological examination of a biopsy specimen revealed a leiomyosarcoma. Laparoscopic resection of the tumor without reduction of the intussusception was performed. The resected line of the proximal intestine was very close to the ligament of Treitz in the present case. Intracorporeal jejunojejunostomy was completed using a delta-shaped anastomosis, wherein anastomosis was performed between the posterior walls of the proximal and distal jejunums after minimal mobilization around the ligament of Treitz. The patient’s postoperative course was uneventful, and he was discharged at 10 days postoperatively. No recurrence has been observed within 2 years after surgery.

**Conclusions:**

We present a case in which a totally laparoscopic surgery for leiomyosarcoma located at the angle of Treitz with jejunojejunal intussusception was performed successfully.

## Background

A leiomyosarcoma (LMS) of the gastrointestinal (GI) tract belonging to soft tissue sarcomas is extremely rare and accounts for approximately 1% of malignant mesenchymal tumors in the GI tract [1], and surgical resection is the only curative treatment option. No report exists on complete laparoscopic surgery of the LMS of the GI tract, especially in the angle of Treitz. Herein, we report a case of jejunal LMS with intestinal intussusception at the angle of Treitz that was successfully treated with laparoscopic resection followed by intracorporeal reconstruction using a delta-shaped anastomosis [2].

## Case presentation

**Fig. 1:**
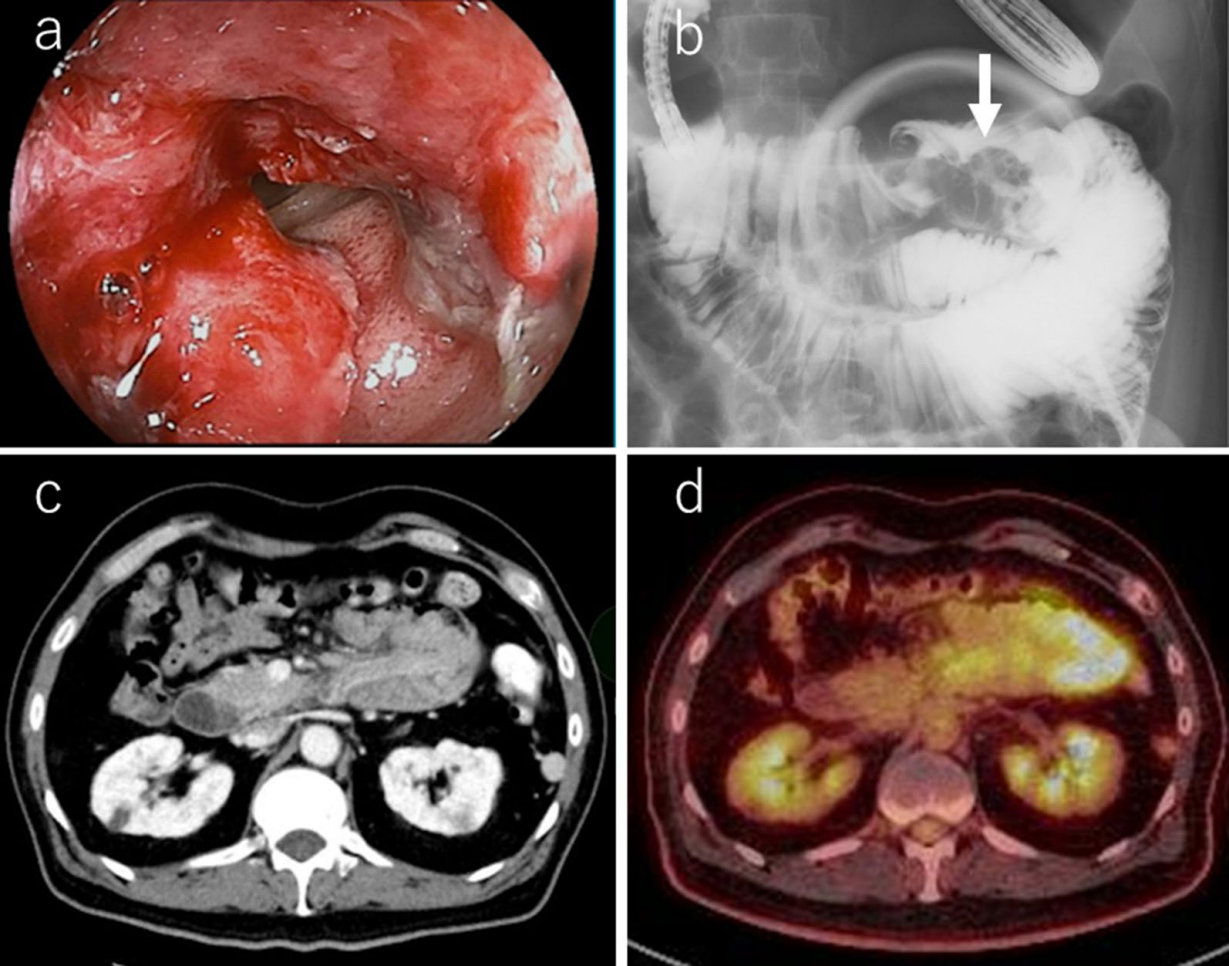
Preoperative imaging findings. **a** Endoscopic findings: enteroscopy revealed an easily hemorrhagic tumor in the jejunum. **b** Findings of the small bowel series using meglumine sodium amidotrizoate: a tumor (white arrow) was found in the jejunum at the angle of Treitz. **c** Contrast-enhanced computed tomography findings: The tumor with intussusception appeared as a reniform shape (pseudokidney sign) longitudinally and demonstrated multilayered concentric rings of mass (target sign) transversely. A tumor with intussusception and regional lymph node swelling was located at the angle of Treitz. **d** Findings of the fluorodeoxyglucose positron emission tomography: this tumor had marked hypermetabolism, showing a standardized uptake value (SUV) max of 8.3, and the swollen regional lymph node showed mild hypermetabolism with a SUV max of 3.7

**Fig. 2:**
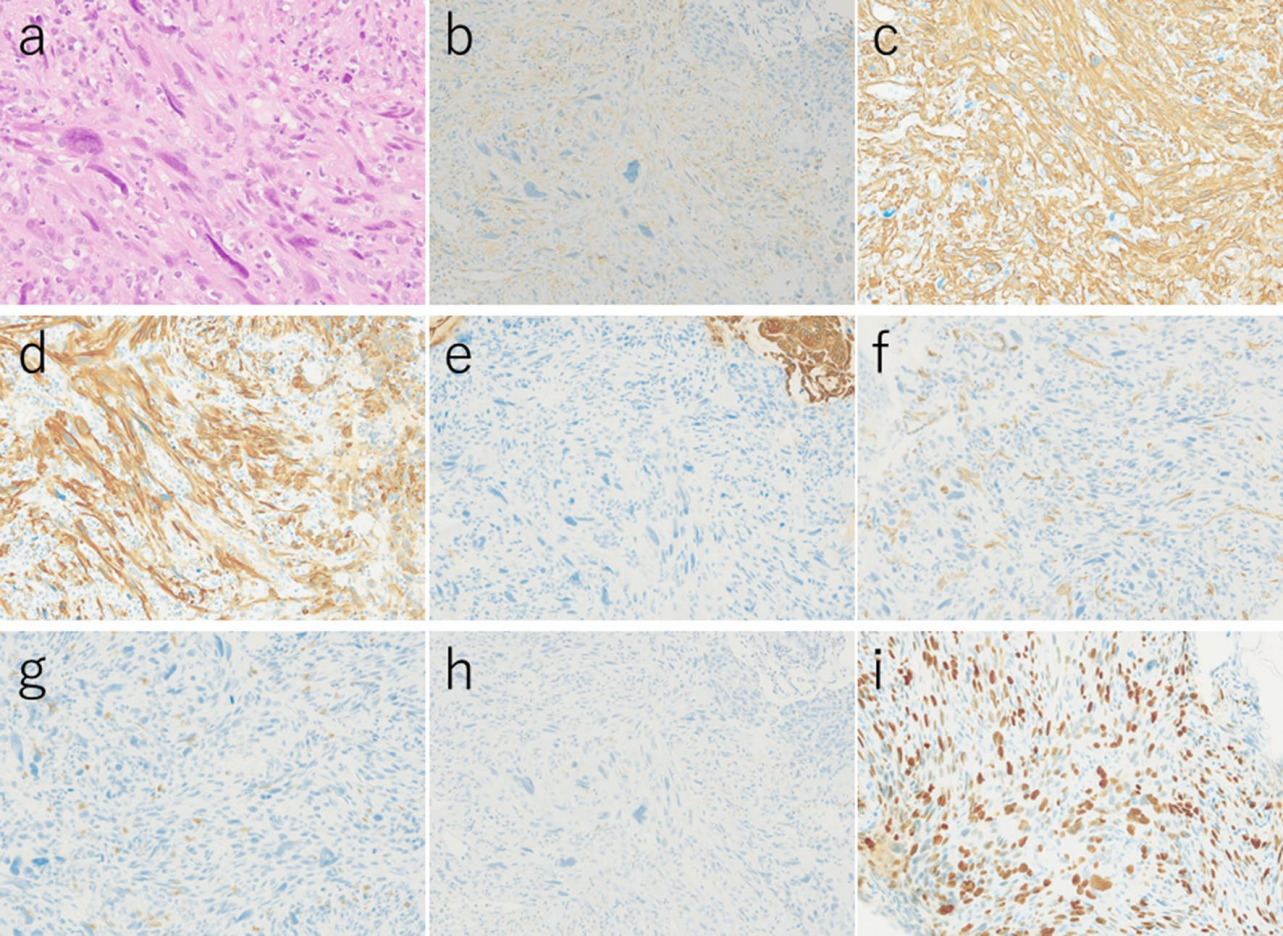
Microscopic and immunohistochemical findings of a biopsy specimen. **a** Hematoxylin and eosin staining. **b** α-Smooth muscle actin. **c** h-Caldesmon. **d** calponin. **e** Vimentin and cytokeratin (AE1/AE3). **f** Cytokeratin (CAM5.2) and CD34. **g** c-Kit. **h** DOG-1 and S-100 protein. **i** Myogenin (the Ki-67 labeling index was approximately 30%)

**Fig. 3:**
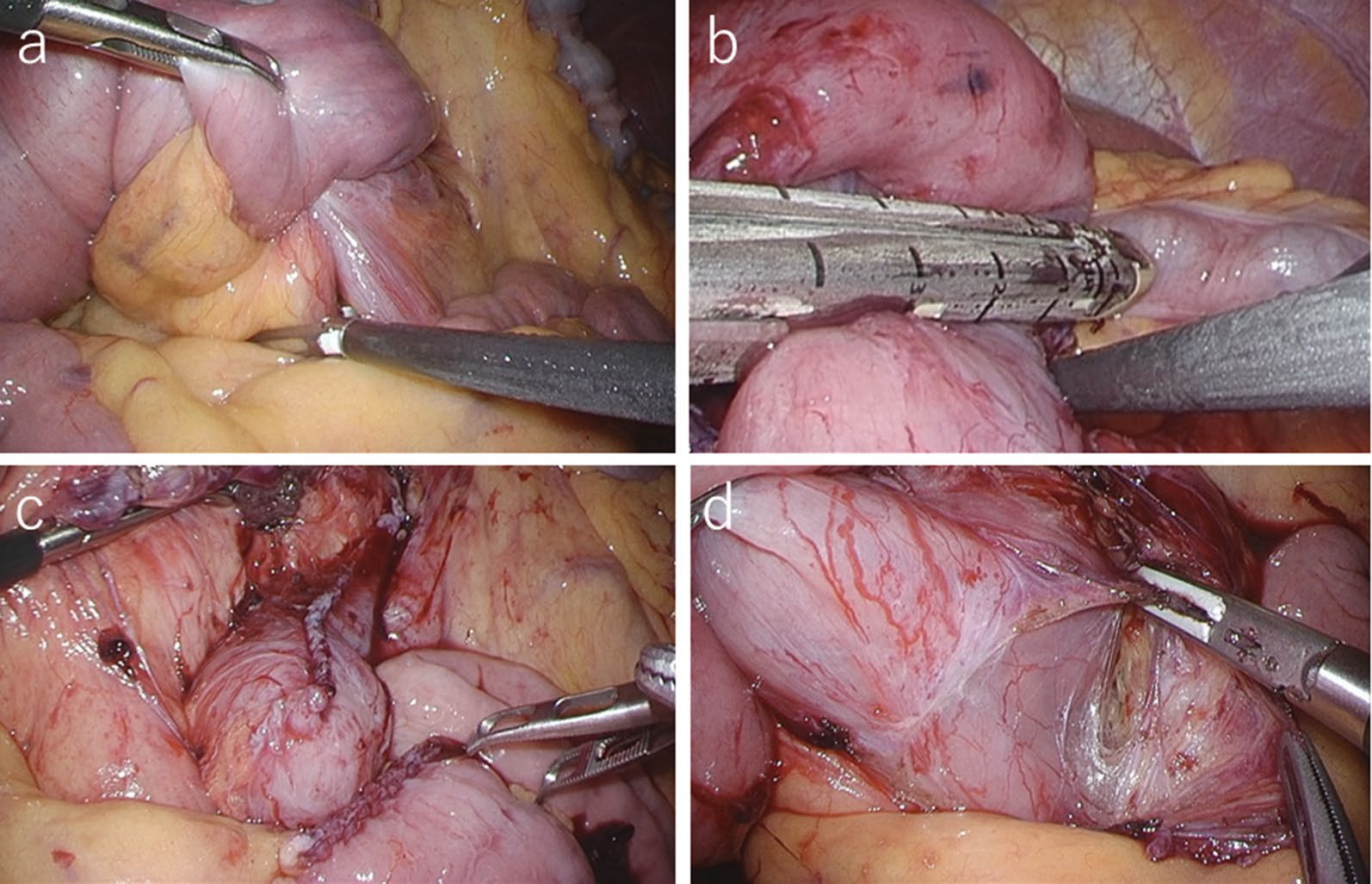
Intraoperative findings. **a** Intestinal intussusception was seen at the angle of Treitz. **b** Transection line of the proximal jejunum extended from the posterior wall to the anterior wall. **c** After resection of the tumor, the residual duodenal stump was short. **d** Proximal jejunal stump was mobilized from the retroperitoneum

**Fig. 4:**
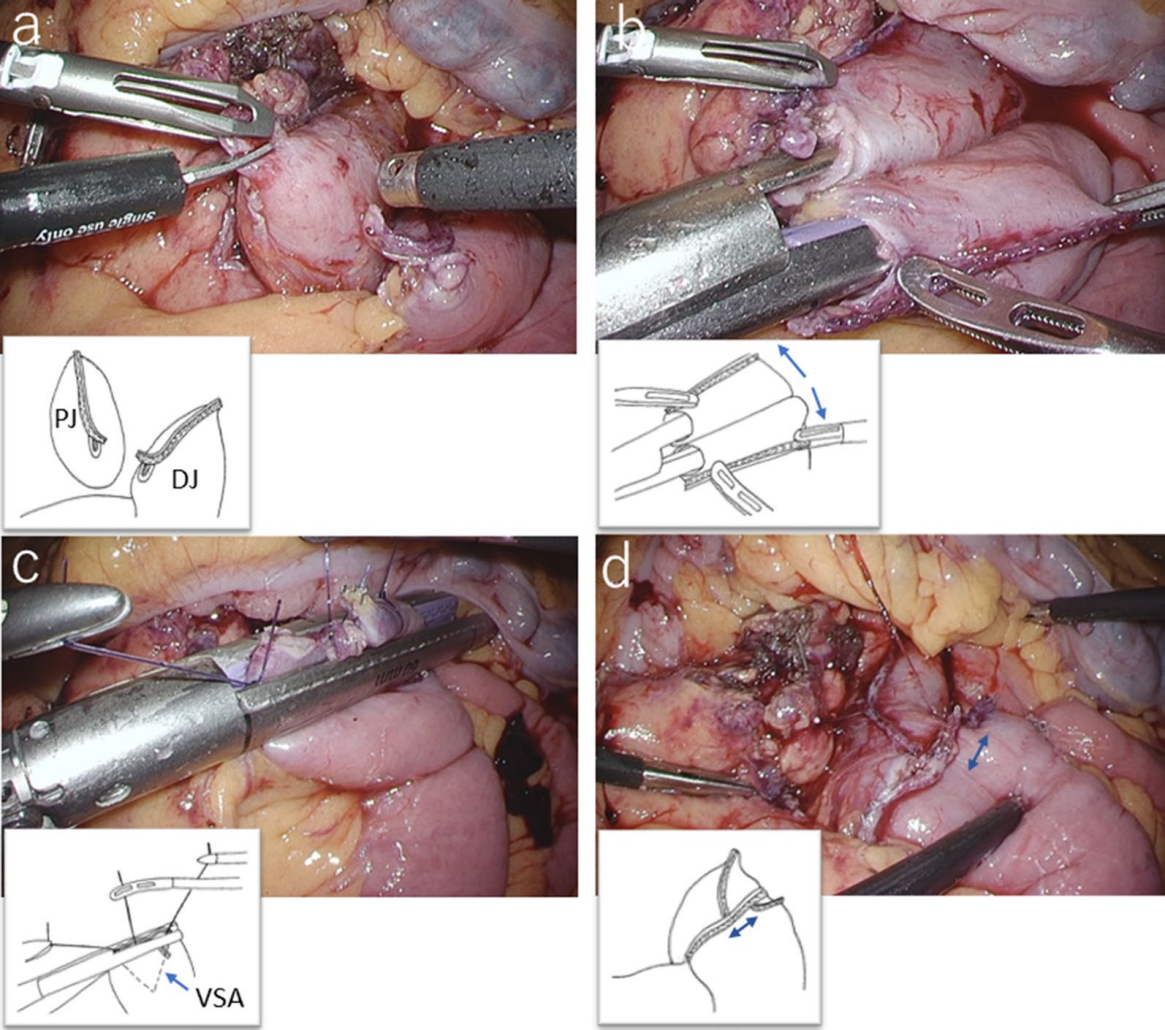
Procedure for creating a delta-shaped anastomosis. **a** Small hole was created on the posterior side of the staple line of the proximal jejunum and on the mesenteric side of the staple line of the distal jejunum. **b** Dorsal walls of the proximal and distal walls of the jejunum were stapled with sufficient distance (arrows) between each other’s staple lines to maintain blood flow. **c** Common entry hole was temporarily closed by three full-thickness sutures while widening the V-shaped anastomosis made by first stapling and then permanently closed with a stapler. **d** Jejunojejunostomy was performed using delta-shaped anastomosis (double arrow: an appropriate gap between the staple lines of the proximal and distal jejunum stumps maintained the physiological axes of the intestinal tract). *PJ* proximal jejunum, *DJ* distal jejunum, *VSA* V-shaped anastomosis

**Fig. 5:**
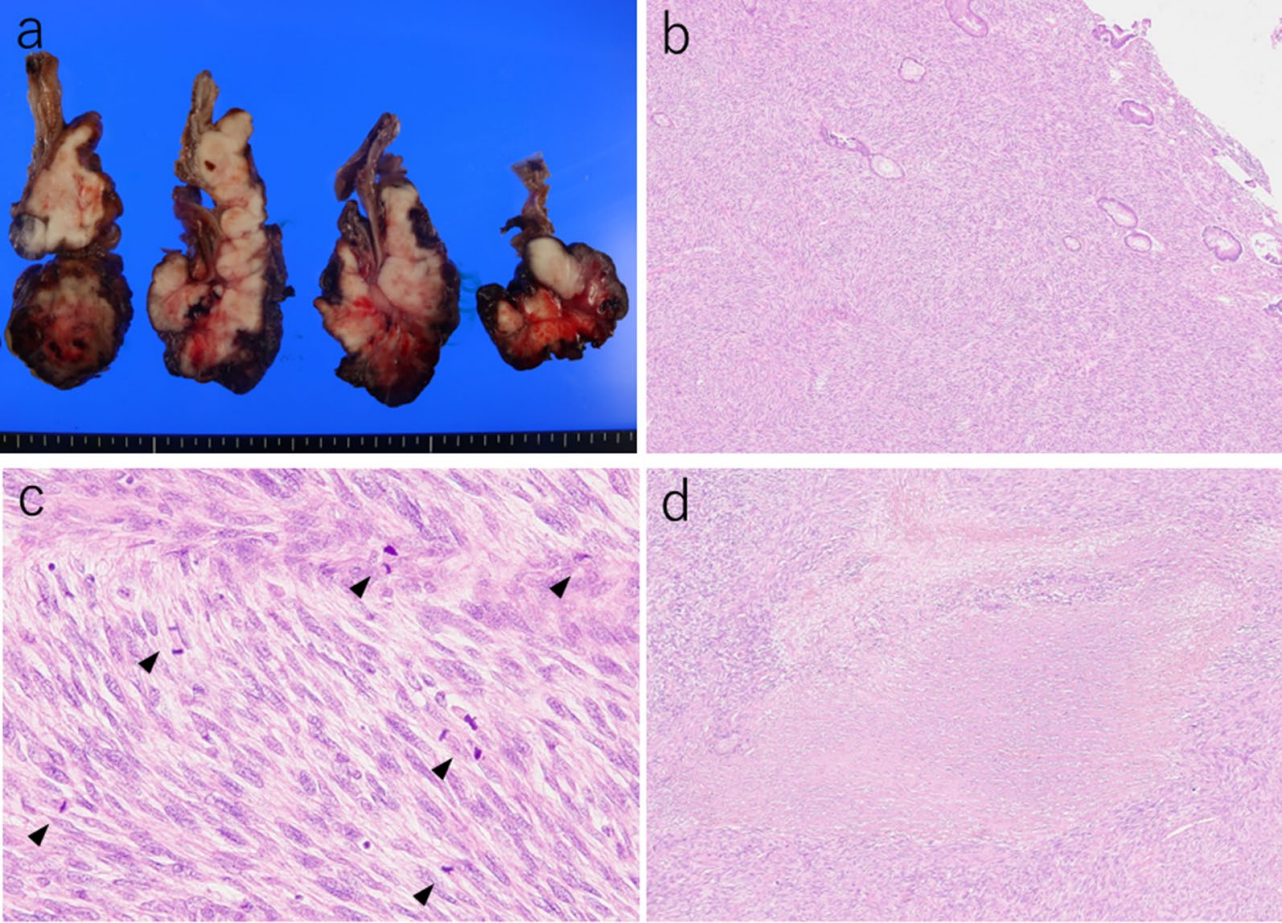
Macroscopic findings of the cross-section and histological findings of the resected specimen. **a** Tumor was a white solid mass (10 × 8 × 5 cm in size) located throughout the jejunal wall. **b** Tumor grew by engulfing the jejunal mucosa. **c** Tumor cells showed a brisk mitotic activity (arrow, > 20 mitoses per 10 high power fields). **d** There were scattered tumor necroses

**Fig. 6:**
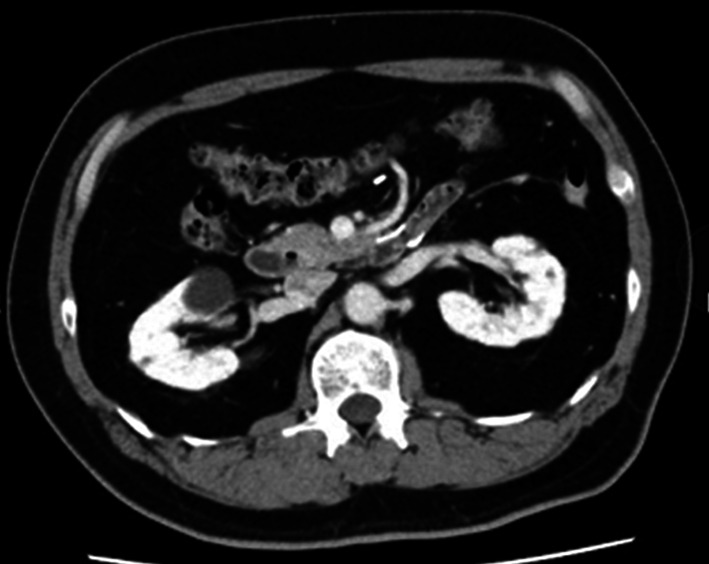
Contrast-enhanced computed tomography findings 2 years after surgery. Neither signs of recurrence nor stenosis has been observed

A 54-year-old man with loss of appetite and fatigue 3 weeks prior was admitted to our hospital due to vomiting and anemia. His medical history included depression and arrhythmia. He had no family history of malignant neoplasia. During presentation, he had tachycardia with a heart rate of 95/min was present, but his other vital signs were within normal limit. Abdominal examination showed normal findings with no palpable mass or tenderness. The blood tests showed a low hemoglobin level (6.2 g/dL) with microlytic anemia, whereas the levels of carcinoembryonic antigen and carbohydrate antigen 19–9 were within the normal range. An esophagogastroduodenoscopy showed no obvious abnormalities. A double-balloon enteroscopy and enterography using meglumine sodium amidotrizoate showed a large circumferential tumor located in the jejunum just beyond the angle of Treitz (Fig. 1a, b). Contrast-enhanced computed tomography from the chest to the pelvis revealed a tumor that equally contrasted with jejunum, inducing intussusception and regional lymph node swelling at the angle of Treitz, although there were no obvious findings of distant metastasis (Fig. 1c). Fluorodeoxyglucose positron emission tomography demonstrated that this neoplasm had marked hypermetabolism, showing a standardized uptake value (SUV) max of 8.3, and the swollen regional lymph node showed mild hypermetabolism with a SUV max of 3.7 (Fig. 1d). Histopathological examination of a biopsy specimen revealed fascicles of spindle-shaped cells with elongated hyperchromatic nuclei showing variably pleomorphism and abundant eosinophilic cytoplasm in the stroma (Fig. 2a). Immunohistochemically, the tumor cells were positive for α-smooth muscle actin (Fig. 2b), h-caldesmon (Fig. 2c), calponin (Fig. 2d), and vimentin, and negative for cytokeratin (AE1/AE3) (Fig. 2e) cytokeratin (CAM5.2), CD34 (Fig. 2f), c-kit (Fig. 2g), DOG-1, S-100 protein (Fig. 2h), and myogenin. The Ki-67 labeling index was approximately 30% (Fig. 2i). Therefore, we diagnosed the patient with a LMS of the jejunum located just beyond the angle of Treitz with intussusception. We considered that the intussusception gradually occurred with tumor growth; thus, elective laparoscopic tumor resection was performed without reducing the intussuscepted jejunum (Fig. 3a–c). The swollen lymph nodes around the tumor were also removed. After extracorporeal extraction of the tumor, an intracorporeal delta-shaped anastomosis was performed in the same manner as that for gastroduodenostomy, as previously described [2]. In brief, the transection line of the proximal jejunum extended from the posterior wall to the anterior wall (Fig. 3b). In contrast, the transection line of the distal intestine extended from the mesenteric side to the antimesenteric side (Fig. 3c). The stump of the proximal side was mobilized by dissecting the ligament of Treitz from the retroperitoneum (Fig. 3d). Then, a small hole was created on the posterior side of the staple line of the proximal jejunum and on the mesenteric side of the staple line of the distal jejunum (Fig. 4a). A 45-mm linear stapler was inserted into both small holes. The posterior walls of the proximal and distal jejunums were put together, and the stapler was closed and fired (Fig. 4b). Then, maximum possible distance was kept between each staple line to secure sufficient blood perfusion in the area between the staple lines (Fig. 4b). The common entry hole was temporarily closed by three full-thickness stitches while widening the V-shaped anastomosis made by first stapling (Fig. 4c) and then permanently closed with one application of a 60-mm linear stapler (Fig. 4c, d) [3]. These procedures produced a torsion-free anastomosis while maintaining the physiological axes of the intestinal tract, resulting in an appropriate gap in the staple line of the common entry hole closure between the staple lines of the proximal and distal jejunum stumps (Fig. 4d) [3]. The operative time and estimated blood loss were 263 min and 180 ml, respectively. The time required for delta-shaped anastomosis was 20 min. Macroscopy of the resected tumor revealed a white solid mass with a size of 10 × 8 × 5 cm located throughout the jejunal wall (Fig. 5a). Histologically, the tumor grew by engulfing the jejunal mucosa (Fig. 5b). The tumor cells showed a brisk mitotic activity (> 20 mitoses per 10 high power fields) (Fig. 5c), and there were scattered tumor necroses (Fig. 5d), indicating that the histological grade of the tumor was 3 according to the French Federation Nationale des Centers de Lutte Contre le Cancer system [4]. All surgical margins were negative for the tumor, and there were no obvious findings of lymphovascular invasion. Furthermore, there were no metastatic findings in all dissected lymph nodes (0/13). The patient’s postoperative course was uneventful, and he was discharged at 10 days after surgery. Neither signs of recurrence nor stenosis has been observed within the 2 years after surgery (Fig. 6).

## Discussion

We experienced a very rare case of LMS in the small intestine at the angle of Treitz, for which laparoscopic resection was performed, followed by delta-shaped anastomosis. In the present case, totally laparoscopic surgery led to a good postoperative course, including quick recovery and no wound infection.

LMS of the GI tract is extremely rare with an incidence compared to gastrointestinal stromal tumor (GIST) of 1:50–65 [1, 5, 6]. Its biological behavior is aggressive, and it has a poor prognosis; the response rates of chemotherapy and targeted treatments such as the administration of tyrosine kinase inhibitors, on LMS have been low [7, 8], and radiotherapy also seems to be unbeneficial due to radio-resistance [9, 10]. Thus, surgical resection has remained the only curative treatment option. Nevertheless, the recurrence rate after surgery has been reported to be high ranging from 39 to 80% [11, 12], and the 5-year overall survival rate was 51.6% [5]. Although surgical resection with negative margins is important, some studies suggest that regional lymphadenectomy did not contribute to the improvement of the long-term outcomes [8, 13, 14].

Although the number of reports on the LMS of the GI tract has been limited, the number of reports of minimal invasive surgery for the LMS of the GI tract have been recently increasing with favorable outcomes. A PubMed search using the key words “leiomyosarcoma” and “laparoscopic surgery” since 1998 revealed six case reports (seven patients) of laparoscopic resection of the LMS in the GI tract [6, 9, 11, 15–17]. Among them, two cases were located in the small intestine, one in the stomach, and the remaining four in the colon. Although the complications in one case had not been described, the remnant cases had no postoperative complications (Table 1). These findings suggest that laparoscopic resection of the LMS in the GI tract could contribute to favorable short-term outcomes. Regarding anastomotic procedures, intracorporeal anastomosis was performed in four cases, except for one case in which the anastomotic procedure was not described. The present case was the only one in which intracorporeal end-to-end anastomosis using a linear stapler (delta-shaped anastomosis) was performed after resecting the LMS in the small intestine. All cases had no lymph node metastasis, and during the observation periods that ranged from 6 to 24 months, no recurrence was observed, except for one who had invasion into the peritoneum and abdominal wall and two who were not described about outcomes (Table 1). These findings indicate that regional lymphadenectomy could not potentially contribute to the improvement of the long-term outcomes [11].

**Table 1:** Case reports of laparoscopic resection of the leiomyosarcoma in the gastrointestinal tract

Case	Author (year)	Age	Sex	Site	Size (cm)	Procedure	Postoperative complication	Lymph node metastasis	Invasion of other organs in pathology	Prognosis
1	Hamm [6] (2013)	45	F	Proximal ileum	8 × 6 × 4.5 cm	Segmental resection and intracorporeal side-to-side anastomosis	–	Not described	–	No recurrence at 6 MAS
2	Bananzadeh [9] (2021)	48	M	Sigmoid colon	8 × 6 × 4.5 cm	Anterior resection and intracorporeal colorectal anastomosis	–	No lymph node metastasis	–	No recurrence at 21 MAS
3	Bananzadeh [9] (2021)	49	M	Descending colon	3 × 4 × 3.5 cm	Left hemicolectomy and extracorporeal anastomosis	–	No lymph node metastasis	+ (Peritoneum and abdominal wall)	Recurrence at 16 MAS
4	Guzel [11] (2016)	87	M	Terminal ileum	5 × 4 × 3.7 cm	Segmental resection and extracorporeal end-to-end anastomosis	–	Not described	–	No recurrence at 12 MAS
5	Yahagi [15] (2019)	46	M	Sigmoid colon	4.2 × 3.7 × 2.8 cm	Sigmoid colectomy and intracorporeal colorectal anastomosis using the double-stapling technique	–	No lymph node metastasis	–	No recurrence at 18 MAS
6	Takagi [16] (2021)	59	M	Proximal stomach	1.8 × 1.5 × 1 cm	Partial gastrectomy and intracorporeal hand–sewn sutures	–	No lymph node dissection	–	No recurrence at 12 MAS
7	Wong [17] (2021)	59	M	Cecum	2 × 2 × 0.7 cm	Right hemicolectomy (the anastomotic procedure was not described)	Not described	No lymph node metastasis	–	No recurrence at 6 MAS
8	Our case (2022)	54	M	Proximal jejunum	10 × 8 × 5 cm	Segmental resection and intracorporeal end-to-end anastomosis using a linear stapler (delta-shaped anastomosis)	–	No lymph node metastasis	–	No recurrence at 24 MAS

*MAS* months after surgery

Contrarily, there have been some reports of totally laparoscopic surgery for tumors of the angle of Treitz [7, 18]. Tanaka et al. and Bracale et al. have reported a case of a GIST at the angle of Treitz that was treated with totally laparoscopic resection, followed by an intracorporeal duodenojejunostomy between the second or third duodenal portion and the jejunum [7, 18]. They considered this procedure as technically demanding due to the anatomical complexity around the ligament of Treitz [7]. Especially, the superior mesenteric artery, superior mesenteric vein, and the confluence of the inferior mesenteric and splenic veins are close by, and the duodenojejunal junction is firmly adherent to the retroperitoneum. Therefore, there is a risk of injuring these major vessels and the intestine during mobilization around the ligament of Treitz [7, 18]. In the present case, an intracorporeal delta-shaped anastomosis was performed. As a result, we could successfully complete the intracorporeal jejunojejunostomy with minimal dissection around the ligament of Treitz in such a short time. To the best of our knowledge, this is the first case of totally laparoscopic jejunojejunostomy using delta-shaped anastomosis after resection of a tumor at the angle of Treitz. Delta-shaped anastomosis has been recognized as a major intracorporeal Billroth I reconstruction procedure after laparoscopic distal gastrectomy, wherein circumferentially full-thickness anastomosis with good blood perfusion is easily created without slack and torsion, similarly to functional end-to-end anastomosis or overlap anastomosis; this technique has been reported to be safe with highly reproducible outcomes [3]. In addition, it seems likely that this procedure is advantageous owing to its minimal requirement of mobilization of the anastomotic intestinal tract as compared to functional end-to-end anastomosis or overlap anastomosis. Recently, Tajima et al. have reported on the application of this procedure in reconstruction after laparoscopic colectomy in the same manner as gastroduodenostomy, with favorable short-term outcomes [19]. This study suggests that the application of the delta-shaped anastomosis in intestinal reconstruction is feasible.

In the present case, we performed tumor resection without reducing intussusception. Although it is controversial whether the intussuscepted intestine is reduced before resection for malignant tumors concurrent with the intussusception, many studies suggest that the reduction procedure can potentially increase the risk of unfavorable outcomes, which are as follows: (1) intraluminal seeding and venous tumor dissemination, (2) perforation and seeding of microorganisms and tumor cells to the peritoneal cavity, and (3) anastomotic complications of the manipulated friable and edematous bowel tissue [20–23]. In contrast, when a reduction procedure is not performed, excessive bowel resection would be required, and as a result, the reconstruction procedure would become more difficult. In fact, the resected line of the proximal intestine was very close to the ligament of Treitz in the present case. Fortunately, we could perform reconstruction by performing a delta-shaped anastomosis, leading to a favorable operative course. Therefore, we consider that resection without the reduction procedure is preferred for malignant tumors concurrent with intussusception, when the length of the intestine is adequate to safely perform anastomosis.

## Conclusions

We present a case in which a totally laparoscopic surgery for leiomyosarcoma located at the angle of Treitz with jejunojejunal intussusception was successfully performed. In the present case report, this experience is too less to prove the procedure efficacy. Therefore, further studies and investigations to assess the safety and long prognosis of laparoscopic surgery for LMS at the angle of Treitz are necessary.

## Data Availability

Not applicable.

## Abbreviations

LMS: Leiomyosarcoma
GI: Gastrointestinal
SUV: Standardized uptake value
GIST: Gastrointestinal stromal tumor
